# Phenotype and genotype of patients with disorder of sex development due to 5α-reductase deficiency

**DOI:** 10.1186/1687-9856-2015-S1-P112

**Published:** 2015-04-28

**Authors:** Vu Chi Dung, Bui Phuong Thao, Nguyen Ngoc Khanh, Can Thi Bich Ngoc, Maki Fukami

**Affiliations:** 1 Department of Endocrinology, Metabolism and Genetics. National Hospital of Pediatrics, Hanoi, Vietnam; 2 National Research Institute for Child Health and Development, Department of Molecular Endocrinology, Tokyo, Japan

## 

A rare form of the 46,XY disorders of sex development (DSD), 5α-reductase deficiency was first described in patients with pseudovaginal perineoscrotal hypospadias, microphallus, and cryptorchid testes in 1974 by Imperato. This undervirilization in the male is due to an alteration in the 5α-reductase type 2 gene (SRD5A2), which encodes for 5α- reductase activity. Our registry of 750 patients with DSD showed no definitive diagnosis in 80% of cases with 46,XY DSD. Our aim is to identify mutations in SRD5A2 gene and to describe phenotype of detected mutative cases. Mutation analysis was performed for genomic DNA extracted from WBC of 10 patients with 46,XY DSD using PCR and direct sequencing. We identified mutations of SRD5A2 gene in two cases. The first case presented with isolated micropenis at birth, two palpable testes in the normal scrotum. Pelvic ultrasound showed no ovaries and uterus, karyotype was 46,XY and SRY was positive. Serum FSH level was 2.4 UI/L; LH level was 0.9 UI/L and testosterone level was 0.4 nmol/l at 8 years of age. A homozygous missense mutation (p.R237G) was identified in the SRD5A2 gene. The second case presented with microphaslus, penoscrotal hypospadias, gonad bilateral in labioscrotal folds. No uterus and ovaries were found by pelvic ultrasound. Karyotype was 46,XY and SRY was positive. A novel homozygous missense mutation (c.659C>T; p.S220L) was identified in the SRD5A2 gene. Mutation analysis of SRD5A2 gene helps to make definitive diagnosis for patients with 46,XY DSD.

